# Three-Dimensional Echocardiography for the Evaluation of the Left Ventricular Outflow Tract in a Patient with a History of Mitral Valve Replacement

**Published:** 2019-01

**Authors:** Maryam Khoshnevis, Ali Hosseinsabet

**Affiliations:** 1 Cardiologist, Tehran Heart Center, North Karegar Street, Tehran, Iran. 1411713138. Tel: +98 21 88029731. Fax: +98 21 88029731. E-mail: khoshnevismaryam@gmail.com.; 2 Associate Professor of Cardiology, Tehran Heart Center, North Karegar Street, Tehran, Iran. 1411713138. Tel: +98 21 88029731. Fax: +98 21 88029731. E-mail: a-hosseinsabet@tums.ac.ir.

Dear editor,

A 46-year-old lady who had a history of a 27-mm St. Jude mechanical mitral prosthesis implantation and tricuspid annuloplasty 8 years previously for rheumatic valvular disease and mitral valve cleaning for mitral valve malfunction as a result of thrombosis 2 years earlier was hospitalized due to increasing dyspnea.

**Figure 1:**
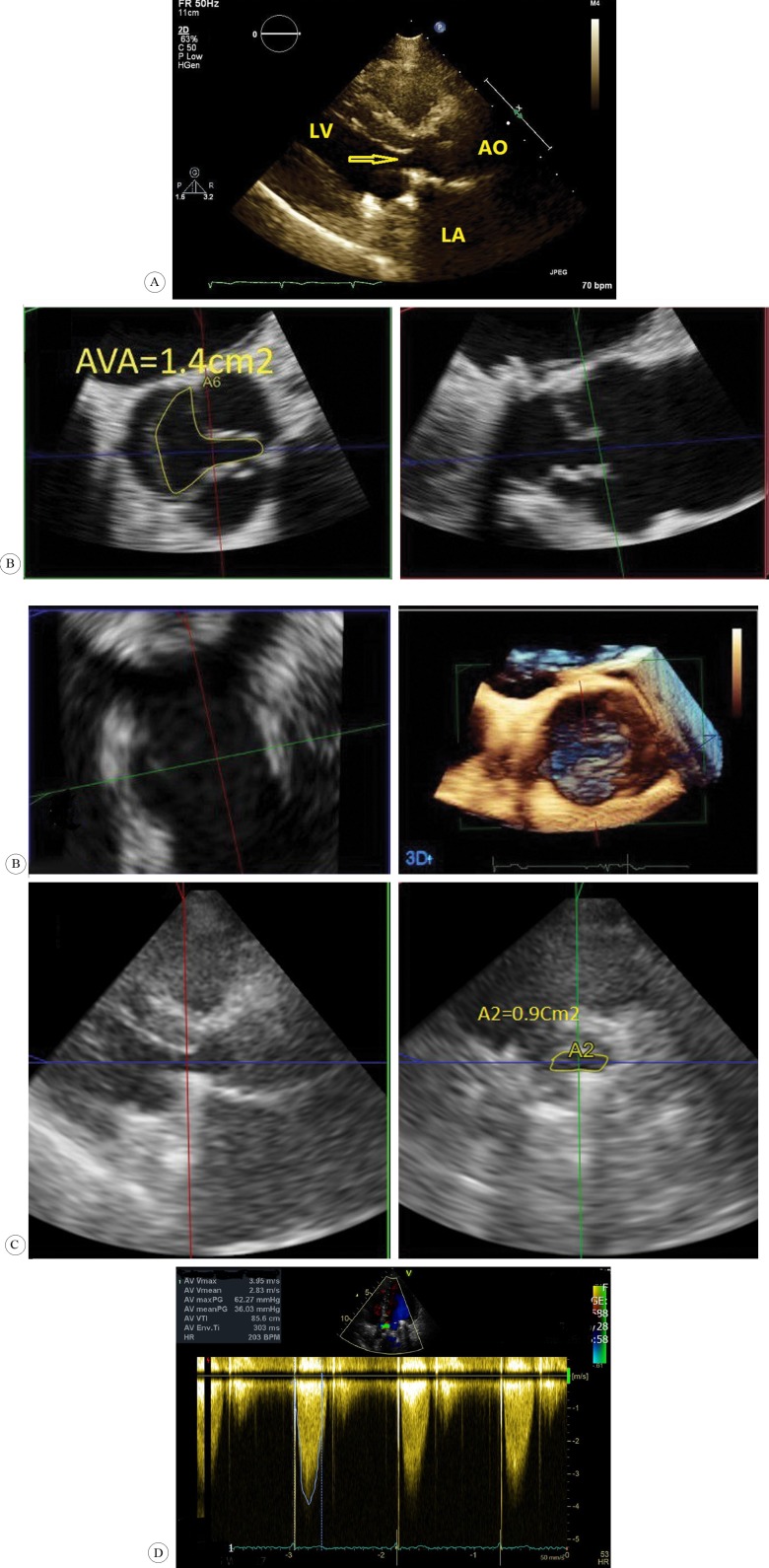
A) Anterior displacement of the mechanical prosthetic mitral valve, resulting in the left ventricular outflow tract (LVOT) narrowing (arrow) in the parasternal long-axis view in transthoracic echocardiography; B) Direct planimetry of the aortic valve in three-dimensional (3D) transesophageal echocardiography in the mid-esophageal short-axis of the aortic valve view; C) Direct planimetry of the LVOT using 3D transthoracic echocardiography in the parasternal long-axis view; and D) The LVOT pressure gradient in the 5-chamber view in transthoracic echocardiography evaluated by continuous wave

AO, Ascending aorta; AVA, Aortic valve area; LA, Left atrium; LV, Left ventricle; LVOT, Left ventricular outflow tract

Physical examination revealed nothing positive except for a systolic murmur at the left sternal border, electrocardiography showed no significant finding, and mechanical mitral valve fluoroscopy was normal. Transthoracic and transesophageal echocardiographic examinations demonstrated a left ventricular ejection fraction of 50%, a mechanical prosthetic mitral valve with a normal leaflet motion and acceptable gradients, no visible paravalvular leakage, moderate tricuspid and aortic valve regurgitation, and no tricuspid stenosis. However, there was significant left ventricular outflow tract (LVOT) obstruction (peak pressure gradient=62 mmHg and mean pressure gradient=38 mmHg). For a further evaluation of the aortic valve and the LVOT region, we conducted three-dimensional (3D) echocardiography at the time of transthoracic and transesophageal echocardiography and found that the aortic valve area by direct planimetry was 1.4 cm^2^ and the LVOT area was 0.9 cm^2^ as a result of the anterior displacement of the mitral prosthesis (Figure 1). The reduced aortic valve area did not explain completely what we obtained in the continuous-wave study, so it seems that the main cause was the LVOT obstruction caused by the anterior displacement of the mechanical mitral leaflet, demonstrated by the direct planimetry of the LVOT in 3D echocardiography. The reviewing of the preoperative transthoracic and transesophageal echocardiographic examinations revealed that there was no aortic valve and LVOT obstruction at that time. The reduced aortic valve area may have been due to a low-flow passing aortic valve because of a significant LVOT obstruction.

The LVOT obstruction after mechanical mitral valve replacement rarely occurs.^1^^-^^4^ If the mechanical valve is not sutured to the mitral annulus exactly, the LVOT obstruction due to the anterior displacement of the sewing ring may occur. The correction of this malpositioning results in the elimination of the LVOT obstruction.^2^ Our patient refused any invasive evaluation or intervention. We recommend that special attention be paid to the LVOT region in the evaluation of patients with mitral valve replacement and 3D echocardiography can be helpful in challenging cases.

***To watch the following videos, please refer to the relevant URLs:***

http://jthc.tums.ac.ir/index.php/jthc/article/view/834/823

Videos 1. Anterior displacement of the prosthetic mitral valve, resulting in the left ventricular outflow tract narrowing in the transthoracic parasternal long-axis view

http://jthc.tums.ac.ir/index.php/jthc/article/view/834/824

Video 2. Anterior displacement of the prosthetic mitral valve, resulting in the left ventricular outflow tract obstruction in the transthoracic parasternal long-axis view with color-Doppler mapping 

http://jthc.tums.ac.ir/index.php/jthc/article/view/834/825

Video 3. Anterior displacement of the prosthetic mitral valve, resulting in the left ventricular outflow tract narrowing in transesophageal echocardiography

http://jthc.tums.ac.ir/index.php/jthc/article/view/834/826

Video 4. Narrow left ventricular outflow tract in cropped 3D transthoracic echocardiography
